# Determination of Trace Metals and Essential Minerals in Selected Fruit Juices in Minna, Nigeria

**DOI:** 10.1155/2014/462931

**Published:** 2014-06-12

**Authors:** A. I. Ajai, S. S. Ochigbo, Z. Abdullahi, P. I. Anigboro

**Affiliations:** Department of Chemistry, Federal University of Technology, Minna 920281, Nigeria

## Abstract

Levels of trace metals and essential minerals in selected fruit juice samples purchased from Minna were determined using atomic absorption spectrophotometer (AAS) and Flame photometer. From the obtained result, Cu, Fe, Mn, Na, and Zn were present in all the samples, while Cd, Pb, and Cr were not detectable in all the samples. Concentrations of K range between 1.31 ± 0.10 and 41.20 ± 0.10 mg/100 mL, Na between 15.47 ± 0.15 and 3.50 ± 0.20 mg/100 mL, Mn between Nd and 0.27 ± 0.08 mg/100 mL, Fe between Nd and 0.90 ± 0.05 mg/100 mL, Cu between Nd-0.60 ± 0.00 mg/100 mL, and Zn between Nd-0.09 ± 0.01 mg/100 mL, respectively. The trace metal levels in all the samples were within permissible limit as recommended by WHO for edible foods and drinks and could therefore be taken to compliment the deficiency of these essential minerals from other food sources.

## 1. Introduction

There has been an increasing trend in the production and consumption of local and imported fruit juices, both in the urban and rural areas of Nigeria and the world in general. The presence of impurities and foreign matter in finished products for human consumption is of great concern because they present health hazards when they exceed beneficial limits. The manufacture of juices requires special attention in terms of purity and the sources of water and its purification are crucial for maintaining quality and safety. Drinking water, fruit juices, and most drinks usually contain small amounts of essential trace elements, which contribute to dietary intakes, and the levels of these elements need to be continually monitored and controlled [1]. Some of these elements are important for the normal functions of the body but when their concentrations exceed an allowable limit [2], they cause acute and chronic poisoning leading to significant illness, reduced quality of life, and even death [3]. The presence of trace metals in water, juices, and different food substances and their health implications have been reported by other workers [4–9].

In a world faced with problem of food scarcity, fruits can act as very good sources of alternative nutrients to compliment the deficiency of these nutrients from other food sources, since they are known to be excellent sources of some essential minerals and vitamins and also contain carbohydrates in the form of soluble sugars, cellulose, and starch [10] and act as good sources of antioxidants [11–13].

In Nigeria, there exist different brands of fruit juices; some are manufactured by registered and recognized manufacturing companies and others by companies not recognized by the food and beverages regulatory agency in the country. Considering the importance of fruit juices to the growth and development of man, there is need to constantly monitor the quality of the product that is being sold to the consumer to ensure that their trace and essential minerals content are within the permissible level that will not be deleterious to health and so compliment the nutrient needs of man, thereby improving on the food security of the country. It is in line with these assertions that this study was set out to find out some trace metals and essential minerals content of some fruit juices sold in Minna metropolis.

## 2. Materials and Methods 

### 2.1. Precaution

To ensure reliability of the results, samples were carefully handled to avoid contamination. All glassware used was soaked in 1 M nitric acid for 48 h and rinsed with ultrapure water. The reagents (nitric acid) were of analytical grades.

### 2.2. Samples Collection

15 fruit juices bearing different brand names were purchased from various shops in Minna. They were grouped into three groups as follows:

 Group 1: canned fruit juices made up of five brands;
 Group 2: packaged fruit juices made up of five brands;
 Group 3: Sachet fruit juices made up of five brands.

### 2.3. Digestion of Fruit Juices Samples

10 mL of concentration nitric acid (HNO_3_) was added to 5 mL of the fruit juice samples in a beaker and the solution was heated on a hot plate in a fume hood for 1 hour. This was then allowed to cool and filtered into a 50 mL volumetric flask and made up to 50 mL mark with distilled water and stored in a polyethylene container prior to analysis.

### 2.4. Analysis of Samples

The digested fruit juice samples were analysed for their trace metals and essential minerals using atomic absorption spectrophotometer (AAS) model 210/211 VGP Buck scientific at Ibadan, while Na and K were analysed using Flame photometer model Jenway PFP-7, UK.

### 2.5. Determination of the Efficiency of the Method

To determine the efficiency of the method, a recovery study was carried out. This was done by spiking a known concentration (0.5 mg/L) of one of the metals of interest into one of the juice samples prior to digestion and the sample was subjected to all the pretreatment steps as other samples and analysed using AAS after pretreatment and digestion. The percentage recovery was calculated using the following expression:

(1) %  Recovery  =Concentration  obtained  (mg/L)−Concentration  added  (mg/L)Concentration  added  (mg/L).

## 3. Results and Discussions

Table 1 shows the result of the mean concentrations (± standard deviation of trace metals and minerals in the different brands of canned fruit juices). They are represented by code name since the objective of the study was to obtain an overview of what was in the market at that point in time. The mean concentrations of sodium in the different canned juices ranged 212.70 ± 0.15–276.70 ± 0.15 mg/L with pineapple fruit juice sample having the highest concentration and mango fruit juice sample the lowest concentration, respectively. Other metals have concentrations ranging 58.80 ± 0.07–194.00 ± 0.20 mg/LK, 1.20 ± 0.02–9.00 ± 0.05 mg/L Fe, 0.20 ± 0.01–0.60 ± 0.01 mg/L Cu, 0.40 ± 0.00–0.90 ± 0.01 mg/L Zn, and 2.20 ± 0.02–2.70 ± 0.08 mg/L Mn, respectively, while Pb and Cr were not detected in all the canned fruit juices studied.

The concentration of Fe, Cu, and Zn in different canned fruit juices ranged 1. 20 ± 0.02–9.00 ± 0.05 mg/L, 0.20 ± 0.01–0.60 ± 0.01 mg/L, and 0.40 ± 0.01–0.90 ± 0.01 mg/L, respectively, with mango fruit juice having the highest concentration of Fe (0.90 ± 0.05 mg/L) and Zn (0.90 ± 0.01 mg/L) and guava fruit juice having the highest concentration of Cu (0.60 ± 0.01 mg/L). It should be noted that all these metals are commonly associated with plants or plant products. But most of the fruit juices do not contain Mn, Pb, and Cr indicating lack of chronic contaminants in the studied fruit juices.

Table 2 shows the concentration of trace metal and minerals in different packaged fruit juices analysed. The major minerals contained were Na and K, with Fe, Cu, and Zn being present in some of the brands. The concentration of Na and K ranged between 154.70 ± 0.15–335.00 ± 0.20 mg/L and 54.50 ± 0.10–412.00 ± 0.10 mg/L with Ribena fruit juice sample having the least concentration of Na (154.70 ± 0.15 mg/L) and Soy good mango fruit juice sample having the highest (335.00 ± 0.20 mg/L) and Happy Hour peach fruit juice having the least of K (54.50 ± 0.10 mg/L) and Five Alive Citrus Burst fruit juice having the highest K of 412.00 ± 0.10 mg/L, respectively.

Table 3 also shows the trace metal and minerals in different Sachets fruit juices studied. It also shows the presence of Na, K, Fe, and Cu with Na and K having higher concentration ranging between 185.70 ± 0.12–275.3 ± 0.10 mg/L Na in Chivita premium fruit juice and Vita vite multivitamin fruit juice and 13.10 ± 0.10–56.10 ± 0.07 mg/L K in Fruit Maid pineapple fruit juice and Vita vite multivitamin fruit juice, respectively. Fe on the other hand ranged Nd-2.50 ± 0.10 mg/L Fe with Chivita premium fruit juice having the highest and Exotic chi orange peach nectar fruit juice the highest. However, Cu was present in trace amount in all the studied Sachet fruit juices and ranged 0.30 ± 0.01–0.50 ± 0.00 mg/L, respectively.

The more toxic trace metals, Mn, Pb, and Cr, were not detected in all the Sachet fruit juice studied indicating their suitability for consumption.

## 4. Efficiency of the Method 

The efficiency of the method was determined by recovery study carried out using one of the samples by spiking by known amount of the metal of interest. From Table 1, for instance, 0.5 mg/L of copper was spiked into a sample of pineapple fruit juice and after pretreatment and analysis a concentration of 0.90 mg/L was obtained. Therefore, from the information, the percentage recovery for copper using the instrument is 80%. This percentage recovery indicates that the instrument has the potential of giving an accurate result. According to European commission [14], recovery of 50–120% is an indication that a procedure adopted for an analysis is an acceptable procedure.

## 5. Discussion 

The concentrations of Fe, Cu, and Zn are very low or completely not detected in some of the studied fruit juices (Table 1). This variation can be attributed to the packaging materials used, while the presence of these metal ions in canned fruit juices in Table 1 can be attributed to the leaching of the canned containers used by the acidic medium of the juices since some of these canned materials are made with these metals such as Zn and Fe.

Manganese which ranged 0.00–2.70 ± 0.08 mg/L in this study is essential metal and a potent neurotoxin. Manganese accumulates in mitochondria and acts as a major source of superoxide, which can oxidize Mn^2+^to the powerful oxidizing agents Mn^3+^. Oxidation of important cell components by Mn^3+^ has been reported as the cause of toxic effect of manganese [15].

Maduabuchi et al. [16] reported cadmium levels of 0.003–0.081 mg/L in canned drinks and 0.006–0.071 mg/L in noncanned drinks and lead levels of 0.002–0.0073 mg/L in canned drinks and 0.092 mg/L in noncanned drinks, respectively, while in this study no lead or cadmium was detected in canned, packaged, and Sachet fruit juices.

Krejpcio et al. [17] reported lead, cadmium, copper, and zinc levels of 0.020–0.46 mg/L, 0.004–0.060 mg/L, 0.047–1.840 mg/L, and 0.063–3.39 mg/L, respectively, in canned and noncanned drinks. Cadmium and lead were not detected in this work.

Generally, in the canned and packaged fruit juice, Cd, Pb, and Cr were not detected indicating nontoxicity of the fruit juices and the suitability of the juices for consumption since some of these metals have been reported to be toxic when present in food even in very minute amount. Comparing the essential mineral content in the various canned fruit juices of Nd-27.67 ± 0.15 mg/L with that reported by Iwegbue et al. [18], of between 0.45–4.00 mg/L Zn and 0.37–45.54 mg/L Fe in mango and apple fruit juices samples collected from Agbor and Warri of Delta state Nigeria, there is a correlation between some of the parameters. Despite the low amount of some of these essential minerals in the studied fruit juices, they can still compliment the deficiency of these minerals in the human diet from other food sources when consumed thereby, helping to improve on the nutritional needs of the individual. The variation between the two studies could be attributed to types of samples, production method and experimental error. Generally, the level of more toxic trace metals (Pb, Cd, and Cr) was not detected or below detectable limit in all the samples analyzed suggesting that the fruit juices are safe for human consumption indicating that they were within the permissible limit that is nondeleterious to health as recommended by WHO [19] for drinking water.

The results of the statistical analysis of different canned fruit juices are studied using Duncan multirange test and one-way ANOVA at 95% confidence level. Table 1 shows cluster of results with no specific brand of canned fruit juice having the highest or lowest of the metal ion analysed. The superscript indicates the differences or similarity between various brands. Those with different superscripts across rows for each brand of fruit juices are significantly different from each other (*P* < 0.05), where a < b < c < d < e.

Similarly, clusters of result were obtained from the statistical analysis of the different Sachet fruit juices (Table 2) with no specific brand having the highest burden of the metal ions with the exception of Vitavite multivitamin fruit juice with highest concentration of Na and K.

Generally, the variation in the statistical result with no particular brand having an overriding property over others is due basically to the different processing method adopted and the various additives added by each manufacturer which brought about change in the concentration of the various metal ions in the studied samples.

## 6. Conclusion

From the results of this study, it can be stated that the studied fruit juices are free of toxic trace metals and the mineral content is within the limit that will not be harmful to the consumer and the studied fruit juices are suitable for consumption in order to compliment the deficiency of the essential minerals from other food sources and improve on the overall nutritional needs of the consumer. We also recommend that those manufacturers using can materials for packaging their juices should ensure that they are well lacquered before use to avert leaching of the toxic materials of the can into the juice.

## Figures and Tables

**Table 1:** Trace metal and mineral content in different canned fruit juices (mg/L).

Metals/samples	Guava fruit juice	Pineapple fruit juice	Mango fruit juice	Apple fruit juice	Ginger fruit juice
Na	224.30 ± 0.15^b^	276.70 ± 0.15^e^	212.70 ± 0.15^a^	266.00 ± 0.10^d^	247.30 ± 0.06^c^
K	116.30 ± 0.15^b^	194.00 ± 0.20^d^	58.80 ± 0.07^a^	133.70 ± 0.15^c^	254.00 ± 0.20^e^
Fe	6.50 ± 0.06^d^	5.10 ± 0.10^c^	9.00 ± 0.05^e^	2.70 ± 0.11^b^	1.20 ± 0.02^a^
Cu	0.60 ± 0.01^c^	0.40 ± 0.00^b^	0.40 ± 0.00^b^	0.20 ± 0.01^a^	0.40 ± 0.00^b^
Zn	0.40 ± 0.01^a^	0.70 ± 0.01^c^	0.90 ± 0.01^d^	0.60 ± 0.00^bc^	0.50 ± 0.01^b^
Mn	0.00 ± 0.00^a^	2.20 ± 0.02^b^	0.00 ± 0.00^a^	0.00 ± 0.00^a^	2.70 ± 0.08^b^
Pb	Nd	Nd	Nd	Nd	Nd
Cd	Nd	Nd	Nd	Nd	Nd
Cr	Nd	Nd	Nd	Nd	Nd

Those with different superscripts across rows for each brand of fruit juices are significantly different from each other (*P* < 0.05), where a < b < c < d < e. Key: Nd: not detected.

**Table 2:** Trace metal and mineral content in different packaged fruit juices (mg/L).

Metals/samples	Five Alive Citrus Burst fruit juice	Happy Hour peach fruit juice	Soy good mango fruit juice	Passion fruit juice	Ribena fruit juice
Na	201.70 ± 0.12^b^	234.00 ± 0.01^c^	335.00 ± 0.20^e^	246.70 ± 0.15^d^	154.70 ± 0.15^a^
K	412.00 ± 0.10^e^	54.50 ± 0.10^a^	191.80 ± 0.09^d^	188.60 ± 0.14^c^	81.30 ± 0.09^b^
Fe	1.10 ± 0.04^b^	0.00 ± 0.00^a^	0.00 ± 0.00^a^	1.80 ± 0.03^c^	0.80 ± 0.02^b^
Cu	0.00 ± 0.00^a^	0.50 ± 0.01^c^	0.00 ± 0.00^a^	0.00 ± 0.00^a^	0.30 ± 0.01^b^
Zn	0.30 ± 0.01^bc^	0.40 ± 0.01^c^	0.30 ± 0.01^b^	0.00 ± 0.00^a^	0.00 ± 0.00^a^
Mn	Nd	Nd	Nd	Nd	Nd
Pb	Nd	Nd	Nd	Nd	Nd
Cd	Nd	Nd	Nd	Nd	Nd
Cr	Nd	Nd	Nd	Nd	Nd

Those with different superscripts across rows for each brand of fruit juices are significantly different from each other (*P* < 0.05), where a < b < c < d < e. Key: Nd: not detected.

**Table 3:** Trace metal and mineral content in different Sachet fruit juices (mg/L).

Metals/samples	Chivita premium fruit juice	Exotic chi orange peach Nectar fruit juice	Fruit maid pineapple fruit juice	Vitavite multivitamin fruit juice	California pineapple fruit juice
Na	185.70 ± 0.12^a^	247.30 ± 0.12^c^	234.30 ± 0.06^b^	275.30 ± 0.10^e^	253.00 ± 0.06^d^
K	43.30 ± 0.06^c^	46.70 ± 0.15^d^	13.10 ± 0.10^a^	56.10 ± 0.07^e^	36.80 ± 0.08^b^
Fe	Nd	2.50 ± 0.10^c^	1.90 ± 0.17^a^	2.40 ± 0.02^b^	2.50 ± 0.05^c^
Cu	0.30 ± 0.01^a^	0.30 ± 0.02^a^	0.30 ± 0.01^a^	0.30 ± 0.01^a^	0.50 ± 0.00^b^
Zn	Nd	Nd	Nd	0.80 ± 0.01	Nd
Mn	Nd	Nd	Nd	Nd	Nd
Pb	Nd	Nd	Nd	Nd	Nd
Nd	Nd	Nd	Nd	Nd	Nd
Cr	Nd	Nd	Nd	Nd	Nd

Those with different superscripts across rows for each brand of fruit juices are significantly different from each other (*P* < 0.05) where a < b < c < d < e; Key: Nd = Not detected.
